# Mental Imagery in Dreams of Congenitally Blind People

**DOI:** 10.3390/brainsci13101394

**Published:** 2023-09-30

**Authors:** Jungwoo Kang, Rita Bertani, Kausar Raheel, Matthew Soteriou, Jan Rosenzweig, Antonio Valentin, Peter J. Goadsby, Masoud Tahmasian, Rosalyn Moran, Katarina Ilic, Adam Ockelford, Ivana Rosenzweig

**Affiliations:** 1 Sleep and Brain Plasticity Centre, Department of Neuroimaging, Institute of Psychiatry, Psychology and Neuroscience (IoPPN), King’s College London, London WC2R 2LS, UK; 2 Department of Philosophy, King’s College London, London WC2R 2LS, UK; 3 Department of Engineering, King’s College London, London WC2R 2LS, UK; 4 Basic and Clinical Neuroscience, IoPPN, King’s College London, London WC2R 2LS, UK; 5 NIHR-Wellcome Trust King’s Clinical Research Facility, King’s College London, London WC2R 2LS, UK; 6 Institute of Neuroscience and Medicine, Brain and Behaviour (INM-7), Research Centre Jülich, 52428 Jülich, Germany; 7 Department of Neuroimaging, Institute of Psychiatry, Psychology and Neuroscience (IoPPN), King’s College London, London WC2R 2LS, UK; 8 BRAIN, Department of Neuroimaging, King’s College London, London WC2R 2LS, UK; 9 Centre for Learning, Teaching and Human Development, School of Education, University of Roehampton, London SW15 5PJ, UK; 10 Sleep Disorders Centre, Guy’s and St Thomas’ NHS Foundation Trust, London SE1 1UL, UK

**Keywords:** dream, congenitally blind, cross-modal plasticity

## Abstract

It is unclear to what extent the absence of vision affects the sensory sensitivity for oneiric construction. Similarly, the presence of visual imagery in the mentation of dreams of congenitally blind people has been largely disputed. We investigate the presence and nature of oneiric visuo-spatial impressions by analysing 180 dreams of seven congenitally blind people identified from the online database DreamBank. A higher presence of auditory, haptic, olfactory, and gustatory sensation in dreams of congenitally blind people was demonstrated, when compared to normally sighted individuals. Nonetheless, oneiric visual imagery in reports of congenitally blind subjects was also noted, in opposition to some previous studies, and raising questions about the possible underlying neuro-mechanisms.

## 1. Introduction

Historically, the term ‘mental imagery’ has been used to refer to depictions and the experience of sensory information without a direct external stimulus, commonly recalled from memory [1,2]. During these representations one re-experiences a version of the original stimulus or some novel combination of stimuli in one’s mind’s eye [1,2]. More recently, it has been shown that individual sensitivity to a particular sensory input may underlie that person’s sensory imagery deficits [1]. In dreams, (oneiric) imagery is thought to arise from the reactivations and manipulations of sensory cortical representations during sleep, although the exact nature of these mechanisms remains uncertain [3,4,5]. Perhaps unsurprisingly, the presence of visual imagery in the mentation of dreams of congenitally blind people has long been a matter of significant controversy [5,6,7,8,9,10,11,12,13,14,15,16]. To date, it is unclear to what extent the absence or loss of vision affects the sensory and pictorial sensitivity for dream construction [15,17], or, more specifically, how it impacts the ability of the nervous system to integrate sufficient sensory information to produce mental images during dreaming.

Arguably, sensory modalities other than vision (e.g., auditory, haptic/tactile, and olfactory) enable adaptive functional development of the occipitotemporal visual system in the absence of visual stimulation early in life [18,19,20,21,22,23,24,25,26,27]. This model is supported, at least *prima facie*, by evidence of cross-modal neuroplasticity of the “blind visual cortex” and its involvement in episodic memory [28], language [18], and in auditory [21,22,27,29] and haptic [30] processing [5].

Moreover, sleep itself, and more specifically, rapid eye movement (REM) sleep [31], appears fundamental for the full development of the visual cortex [4,32], and, therefore, of mental imagery [4,33,34,35]. Notably, Eagleman and Vaughn have recently proposed that the circuitry underlying REM sleep serves to selectively amplify the visual system’s activity periodically throughout the night, allowing it to defend its territory against takeover from other sensory inputs [36]. It has also been argued that, during the distinct microstates of REM [37], phasic brain-state co-ordination leads to transient differential coherence with hippocampal and other wider thalamo-(visuo)cortical regions [38]. In turn, this may also ensure attentional shifts that ‘reset’ mnemonic processing frames and enable oneiric conscious experiences [39], including discrete epochs of the generation of visual-like mental representations during REM sleep [3,37,40]. With this background, it is of note that congenitally blind people show significantly reduced, or fully absent, rapid eye movements during sleep [8].

Nonetheless, over the years, it has been reported that congenitally blind people can, and do, experience oneiric visuo-spatial imagery in a way that is similar to sighted individuals [5,41,42,43]. In keeping with this, significant negative correlations between the visual activity index (defined by performing a quantitative analysis of dream content, also see [42]) and occipital alpha power have been demonstrated during REM’s dream mentation in congenitally blind subjects [43]. This is largely in line with reduced or blocked alpha power over the occipital cortex, commonly associated with visual imagery in normally sighted people [44,45,46]. Strikingly, some congenitally blind subjects have also been able to represent the visual content of their dreams in accurate drawings, if somewhat less detailed and slightly more symbolic and archetypal, similar to those of sighted controls [42].

Thus, with the background of this ongoing debate [15,16,43,47], we set to investigate the presence and nature of oneiric visuo-spatial impressions by analysing 180 dreams of seven congenitally blind people identified from the online database DreamBank (http://www.dreambank.net/, accessed on 1 May 2021) [48]. We predict nonmetaphorical visual keywords to be significantly less frequent, and nonmetaphorical auditory, haptic, gustatory, and somatosensory keywords to be significantly more frequent in the dream reports of the congenitally blind group, compared to the sighted control group. 

## 2. Methods and Materials

The DreamBank [48] collection is a distinct database of over 20,000 dream reports (Supplement). Its dream reports have been predominantly collected during the last century, preceding the global availability of digital media. Thus, arguably, they may be more devoid of its hypothesised corruptive globalising effect [49], which could impact individual’s dream mentation and subjects’ memories of personally experienced events [50]. All DreamBank participants gave informed consent, and all methods were carried out in accordance with relevant UK and international guidelines and regulations.

### 2.1. Dream Selection

Altogether, 180 dreams of seven congenitally blind subjects were identified in the DreamBank [48] (Table 1). In this study, all participants self-identified as white (U.S.) (also see https://dreams.ucsc.edu/Library/fmid4.html, accessed on 1 May 2021).

Specifically, six congenitally and totally blind subjects were initially identified from a larger DreamBank series of dreams, collected in the 1990s from visually impaired men and women (*Series 1*) [48]. An additional nineteen dreams were then sourced from one congenitally and totally blind participant who was interviewed in late 1940s (*Series 2*).

The normative sample of dreams from the control subjects, also recorded in the past century, were collected from the DreamBank, as previously described [51] (also see Supplement). Overall, 981 dreams from normally sighted gender-matched controls from the DreamBank’s *Series 3* (490 dreams from female subjects) and *4* (491 dreams from male subjects) were identified for the purposes of the statistical analyses [51].

### 2.2. Dream Analysis

The modified dream content analysis was conducted, as previously described [10,14]. More specifically, the relevant dream report series from the DreamBank was selected and a set of sensory keywords belonging to seven predetermined categories was chosen (Table 2) [10,14].

Subsequently, utilising the DreamBank software [48], the sensory content of 180 dreams of congenitally blind and 981 normally sighted subjects was analysed and compared (for more in-depth description, please refer to Supplement).

Prior to the analysis, selected dream reports were quality scanned to exclude those reports in which keywords were used metaphorically, or not in a strictly sensory context; for example, a dream report mentioning “a little gift” was included, as “little” is a size indicator in this case, while reports containing phrases such as “I was a little startled” were excluded. Furthermore, keywords not directly related to the sensations of the subject reporting the dream were not considered in the analysis; for instance, “my left rib hurt” was included, while statements such as “they were not hurt” or “he was in pain” were not included. Afterwards, for each subject group (congenitally blind versus sighted controls), we compiled the total number of dream reports in which any of the categories’ keywords were used in a strictly sensory and self-referential way. This compilation was conducted by two independent investigators (J.K. and R.B.) in a double-blind manner; eventual discrepancies were discussed with a third investigator (K.I.), and rectified accordingly prior to the statistical analysis (Supplementary Table S1).

**Figure 1:**
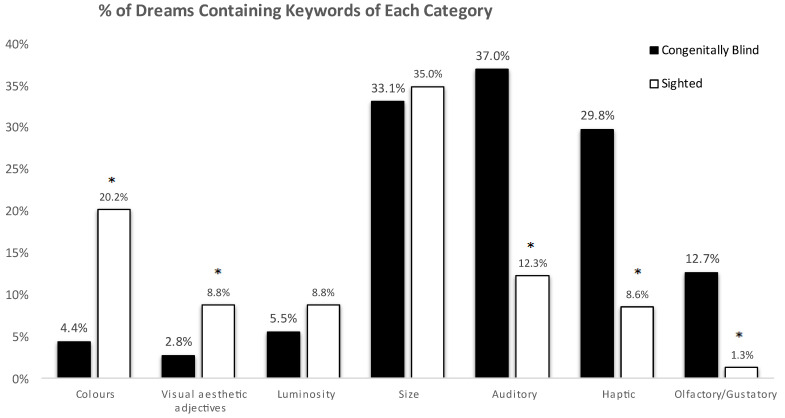
Oneiric sensory impressions in congenitally blind versus sighted controls. Keyword frequency is reported in percentages above the bars for each category. Significance values (*p*) from the chi-squared tests are also reported above the bars. * Denotes *p* < 0.05.

Statistical analysis was conducted with the Statistical Package for the Social Sciences (SPSS) Statistics 26 (IBM Corp., New York, NY, USA). A chi-square test for independence was calculated comparing the occurrence of each keyword’s frequency, for each category, between the dreams of the congenitally blind and those of the normally sighted controls (Figure 1; Table S1).

Statistical significance was set at an alpha of 0.05. 

## 3. Results

Congenitally blind subjects were shown to use words indicating auditory (37.0% versus 12.3%; *p* < 0.001), haptic (29.8% versus 8.6%; *p* < 0.001), gustatory, and olfactory (12.7% versus 1.3%; *p* < 0.001) sensations significantly more frequently when describing their dream content in comparison to normally sighted subjects (Figure 1; Supplementary Table S1). 

In our study, congenitally blind subjects were shown to use visual adjectives such as colours (4.4% versus 20.2%; *p* < 0.001) and (visual) aesthetic judgments [52] (2.8% versus 8.8%; *p* < 0.001); however, their use of these adjectives was present significantly less frequently than in their sighted counterparts. 

No significant difference between the two groups was found for the categories of size (19.3% versus 18.8%; *p* = 0.837), and, interestingly, correspondingly, no significant difference was observed in the category of luminosity (7.7% versus 11.3%; *p* = 0.123).

Additionally, a previously unpublished excerpt from an interview with a congenitally blind subject, where she discusses a dream in which she perceived the colour white, conducted in the late 1940s, is also shown (Table 3) [48].

## 4. Discussion

In keeping with previous studies, we demonstrate a higher presence of auditory, haptic, olfactory, and gustatory sensation in dreams of congenitally blind people, when compared to normally sighted individuals [16,17]. Our report of oneiric visual-like imagery in congenitally blind subjects (Figure 1; Table 3), however, challenges the negative findings in the majority of previous studies [16,17]. On the other hand, our results appear to be in keeping with two studies that have demonstrated (oneiric) visual-like imagery in congenitally and totally blind subjects lacking any previous visual perception or experience [17,41,43].

We also report, for the first time, an excerpt from an interview with a congenitally blind woman (Table 3; DreamBank [48]). Her elaboration of oneiric visual-like experiences is in broad agreement with other anecdotal reports where subjects refute common understanding that their visual-like imagery may reflect merely metaphoric [10,47] or mental representations with preserved spatial and metric properties [43]. Some mechanistic understanding has been gained from the research in the field of lucid dreaming, where lucid dreaming is defined as an experience of achieving conscious awareness of dreaming while still asleep [53]. In lucid dreamers, different spatio-temporal EEG features, with distinct oneiric narrative and imagery, have been demonstrated depending on whether dreams were spontaneous or induced (e.g., by visual stimulation or presleep suggestion) [54]. The former have been linked with increased activity in areas associated with an increased level of visual attention and executive memory processing, with the latter predominantly demonstrating a significant increase in gamma activity in the frontal lobes [54]. However, it remains unknown if lucid dreaming exists, and if so, whether it is less or more prevalent in congenitally blind dreamers. None of the dreams’ narratives in this study suggested lucid dreaming.

Given the period when the dreams analysed in this study were collected, i.e., some stemming from as early as the mid-20th century, it is of interest to consider whether experience and cultural beliefs may have impacted visual imagery and dreaming of our participants [55]. For instance, Schwitzgebel (2002) reported a surprising inconsistency in the results of the earlier and later studies of dreams, with research conducted in the early 20th century consistently demonstrating dreaming in black and white [56]. However, this trend abruptly disappeared in the 1960s, presumably with the advent of colour TV and other media [55]. More recently, it has been suggested that vividness of dream experiences, including experience of colours, may predominantly depend on the intensity of the brain activity in distinct neurocircuitry [57]. However, how distinct anatomical and physiologic processes of the congenitally blind brain may affect this process remains an important unanswered question, with some initial insights gained from neuroimaging studies (for further in-depth systemic review, please refer to [5]).

Historically, it has been recognised that the major experimental conundrum in delineating processes that may underlie any such visual imagery predominantly reside in the limited objectivity of otherwise highly personal and subjective dream reports. Similarly, the overwhelming neurophysiologic complexity of the visual system presents further hindrance [58]. For example, the visual system comprises multiple parallel and interacting processing pathways in the brain that relate and process neural information on form, motion, and colour [43,58]. However, it is uncertain whether there is anatomical separation between the visual cortical areas serving visual imagery and those serving visual perception [43]. Over the years, some neuroscientists have proposed that the regions used for visual imagery present a subset of those engaged in perception, whilst others have maintained that the regions subserving visual perception and imagery are the same (please see [43]). In summary, to date, there remains ambiguity over how these separate pathways are brought together into a single image, and whether the reevoking of images inevitably activate all of them on all the hierarchic levels [43,58,59].

Another interesting possibility could be that, arguably, in a theoretical parallel to Jungian’s notion of archetypal symbols (e.g., protoconsciousness and oneiric primordial images) [60], the eccentric genetic wiring of our early visual cortex [27] supports a possibility of elementary (primordial) ‘visual-like’ or *homoiōma* (“likeness”, in Ancient Greek) neural representations that are inbuilt a priori and onto which other sensory modalities feedback nonvisual and potentially predictive information. If this is indeed the case, this, in turn, would then enable a typical spatio-temporal organization of early visual areas by eccentricity [58] to develop even in the life-long absence of vision [36]. Moreover, such a notion would arguably also explain the striking ability of congenitally and totally blind subjects to draw symbolic representations of various visual images [41] in eerie likeness to those drawn by normally sighted subjects. Somewhat analogous hypotheses have been advanced in the past to explain the protracted language acquisition in autistic individuals in comparison to those with neurotypical development, and are in line with the notion of Hebbian correlation learning in neuroanatomically structured networks which yield distributed circuits binding action and perception information [59]. Perhaps relatedly, Pascual-Leone and Hamilton (2001) have argued that the human brain may operate as an inherently metamodal network, where distinct brain regions may execute a given function or computation regardless of sensory input modality (please refer to [61]).

The existence of *homoiōma* could also be reasoned by the demonstrations of cross-modal neuroplasticity, as evidenced by neuroimaging [5,20,26,62] and sensory substitution [63,64,65] studies [5]. For instance, it has been recently argued that the creation of new connections between the occipital cortex and areas of the brain involved in auditory or haptic processing, and/or the unmasking of existing connections, which are normally inhibited in the presence of vision [25,66,67], may, in the blind, enable integration of nonvisual sensory inputs to generate any such visuo-spatial images [5]. Moreover, parts of the occipital cortex, such as the V1 region, have been shown to undergo cross-modal plastic adaptation in the congenitally blind, and to contribute to nonvisual processing [18,23,25]. However, other occipital areas—such as the extrastriate body area [19], the lateral occipital tactile-visual area [68], and the fusiform and inferior temporal gyri [26] maintain the higher-order, multisensory integration functions that they have in the sighted, and, therefore, presumably at least in part, may contribute to the formation of our reported oneiric *homoiōma*.

Finally, despite obvious limitations of our small cross-sectional study that compared individuals from different time eras, sex/gender, and ages, hence, restricting claims of any causality, we propose that our findings are supportive of the presence of *homoiōma*, or oneiric visual-like imagery in congenitally blind people. However, it remains that other equally plausible alternative explanations cannot be currently excluded, including those arguing for amodal spatial representations in visual cortices of congenitally blind people [69], and those stating that visual-like imagery in the dream reports of blind people may only be understood in metaphorical terms [16]. Nonetheless, it is hoped that our findings will support the growing calls for multicentre and multimodal imaging studies of dreaming and sleep physiology in congenitally blind people. Deciphering the mechanistic nature and the genesis of *homoiōma* may open new possibility in the utilisation of neuroplasticity and its potential role for treatment of neurodisability. 

## Figures and Tables

**Table 1:** Sociodemographic data for the congenitally blind subjects (DreamBank) [48].

Dreambank CODE	Sex	Age	Years of Education	Occupation	Nature/Degree of Blindness	# of Dream Reports
1	F	32	18	Unemployed	C/T	10
2	F	52	12	Envelope stuffer	C/T	37
3	F	44	18	Factory worker (retired)	C/T	32
4	F	44	13	Medical transcriptionist	C/T	9
5	M	45	16	Human resources management	C/T	61
6	M	46	12	Small engine repairs	C/T	12
7	F	18	13	College student	C/T	19

*Abbreviations*: C/T: congenitally blind with no residual vision of any kind; F: female; M: male.

**Table 2:** Keywords used to analyse dream content on DreamBank, divided in seven categories.

Colours	^white^ or ^black^ or ^gold^ or ^silver^ or ^copper^ or ^bronze^ or ^red^ or ^green^ or ^orange^ or ^violet^ or ^purple^ or ^blue^ or ^yellow^ or ^gr[ae]y^
Aesthetic adjectives	pretty or beaut- or gorgeous or handsome or ugly or disgust or attractive
Luminosity	dark or bright or ^light^ or ^lit^ or ^shin(ing|e|ed)^ or illumi or ^sun(^|ny)^
Size	^big(|ger)^ or enormous or huge or ^long^ or ^larg(e|er)^ or ^gi(ant|gantic)^ or ^ta(ll|ller)^ or ^smal(l|ler|lest)^ or t[i|ee]ny or little or ^thi(n|nner|nnest)^
Auditory	^hea(r|rd|ring)^ or sound or ^lou(d|dly|der)^ or ^quiet^ or nois
Haptic/Touch	^touc(h|hing|ed)^ or ^fe(el|lt)^ or ^smooth^ or ^soft^ or ^co(ol|ld) or ^h(eat|ot)^ or ^pai(n|ful)^ or ^hurt^ or ^warm
Olfactory/Gustatory	smel(l|t) or scent or tast(y|e)

**Table 3:** Extract from a representative interview with a congenitally blind college student (DreamBank [48]).

**Dream**	“[…] We went over to a table that was up against the wall, at one end of the studio. The top was covered by a white chiffon tablecloth, very voluminous. It was a gorgeous thing, very soft and full and beautiful. And on the table were two big silver candelabras with candles in them, and I think they were lit. Neither R. nor I were content with the way the tablecloth was arranged, so while we waited for the music to come on, we went over to the tablecloth to rearrange it in nicer folds […]”
Q:	**Do you think R. told you that the tablecloth was white?**
E:	No. I just knew it and I had a visual impression of white which I can’t describe except that it was just devoid of any darkness, no color.
Q:	**Do you often have this sensation?**
E:	No, it’s just as unusual as having a color impression, for me, that is. Actually, of course, I never have any idea of dark and light, neither in the day nor in the night. It’s just nothing at all, but this was a real visual impression of white, at least it was to me. It may just be my conception of white, but there it was.
Q:	**What about the candelabra, did you have an impression of the color silver or do you mean you knew it was of the metal silver?**
E:	Well, I knew it was silver metal because it was very smooth to touch, but I also had the impression of silver and the way I know silver is that it’s like white only shiny.

## Data Availability

All data that support the findings of this study are available and open source at the DreamBank [48].

## Supplementary Materials

The following supporting information can be downloaded at: https://www.mdpi.com/article/10.3390/brainsci13101394/s1, Table S1: Oneiric sensory impressions/words used in dreams of congenitally blind vs. sighted controls.
