# Supplementary material for: Mental Imagery in Dreams of Congenitally Blind People
Source: Brain Sci. 2023 Sep 30;13(10):1394. doi: 10.3390/brainsci13101394 (PMC10605848; doi:10.3390/brainsci13101394)
Supplement: Supplementary file 1 [file brainsci-13-01394-s001.zip › brainsci-2505110-supplementary.pdf]

Supplement to: **Mental Imagery in Dreams of Congenitally Blind People**

Methods

The DreamBank[1] collection (https://www.dreambank.net/) is a remarkable and unique open database of over 20,000 dream reports, collected from a variety of different research studies, from people of ages 7 to 74 years old. It can be analysed using the search and statistical programs in-built into the site and generously maintained by Dr Adam Schneider and Professor G. William Domhoff (*Psychology Department, UC Santa Cruz, USA*).

For the purposes of our study, we used the normative sample of dreams from the normally sighted control subjects[2]. More specifically, from Dreambank's *Series 3* (490 dreams from female subjects) and *Series 4* (491 dreams from male subjects), the dream reports were collected in the following way, as previously reported[2]: from each of 100 male and 100 female series, five out of 12-18 dream-content reports were randomly selected, for a total of 1000 dreams. Nineteen of these selected dreams were not stored on Dreambank, leaving a total number of combined 981 (490 + 491) reports from *Series 3 and 4*. Finally, as previously described, only those dream reports with more than 50 and less than 300 words in length were included in the final selection[2].

Once the dreams were identified, for each subject group (congenitally blind *versus* sighted controls) and sensory category, JK and RB classified dreams in a double-blind manner as 'included' if the sensory category's keywords was used in a strictly sensory and self-referential way, and 'excluded' otherwise. Specifically, dreams were excluded if keywords were used metaphorically, or not in a strictly sensory context; for example, a dream report mentioning "a little gift" was included, as "little" is a size indicator in this case, while reports containing phrases such as "I was a little startled" were excluded. Furthermore, keywords not directly related to the sensations of the subject reporting the dream were not considered in the analysis; for instance, "my left rib hurt" was included, while statements such as "they were not hurt" or "he was in pain" were not included. Dream categorisation for all sensory categories was reviewed by KI, with

subsequent discussion with JK and RB to resolve any discrepancies. Finally, subtracting the number of dreams which were 'included' for each category from the total number of dreams, the number of dreams which did not include the sensory categories was determined and the final data table for statistical analysis was generated. Categorical independent variable was subject group (blind versus sighted), whilst continuous independent variables were frequency of colour, visual aesthetic, luminosity, size, auditory, haptic, and gustatory/olfactory keywords in the dream content reports of each subject group.

Overall, there was a good agreement in dream classification between JK and RB, with discrepancies only arising in the 'luminosity' and 'size' sensory domains. For the dreams containing the 'luminosity' keywords, there were 12 disagreements for 140 dreams (8.6%), and for 'size', there were 23 disagreements for 487 dreams (4.7%).

Results

Table S1. Oneiric sensory impressions/words used in dreams of congenitally blind vs. sighted controls. Significance values (*P*) from the Chi-squared tests are reported.

	**Congenitally Blind**			**Sighted Controls**			$\chi^2 - test$			
	Included	*Excluded*	$\frac{\text{included}}{\text{total}}$	*Included*	*Excluded*	$\frac{included}{total}$	ϕ	χ^2	*df*	*P*
**Colour**	8	173	4.4%	198	783	20.2%	0.15	26.0	1	**<0.001**
**(Visual) Aesthetic Adjectives**	5	176	2.8%	86	895	8.8%	0.08	7.6	1	**0.006**
**Luminosity**	10	171	5.5%	86	895	8.8%	0.04	2.1	1	0.146
**Size**	60	121	33.1%	343	638	35.0%	0.14	0.2	1	0.637
**Auditory**	67	114	37.0%	121	860	12.3%	0.24	68.6	1	**<0.001**
**Haptic**	54	127	29.8%	84	897	8.6%	0.24	66.1	1	**<0.001**
**Olfactory and Gustatory**	23	158	12.7%	13	968	1.3%	0.24	65.9	1	**<0.001**

References

1. Schneider A, Domhoff GW. The Quantitative Study of Dreams. 2021; http://dreamresearch.net/. Accessed 15/02, 2021.
2. Hall CS, Van De Castle RL. *The content analysis of dreams.* East Norwalk, CT, US: Appleton-Century-Crofts; 1966.
